# The gut microbiota-obesity axis in the pathogenesis and prognosis of breast cancer

**DOI:** 10.1080/07853890.2025.2611203

**Published:** 2026-01-07

**Authors:** Huiyue Zhang, Yue Wang, Benyi Ning, Yiwen Wang, Tao Sun, Junnan Xu

**Affiliations:** ^a^ Department of Breast Medicine 1, Cancer Hospital of China Medical University, Liaoning Cancer Hospital, Shenyang, China; ^b^ Department of Pharmacology, Cancer Hospital of China Medical University, Liaoning Cancer Hospital, Shenyang, China; ^c^ Department of Oncology Medicine, Key Laboratory of Liaoning Breast Cancer Research, Shenyang, China; ^d^ Department of Breast Medicine, Cancer Hospital of Dalian University of Technology, Liaoning Cancer Hospital, Shenyang, China

**Keywords:** Breast cancer, gut microbiota, obesity, immunity, prognosis, dysbiosis

## Abstract

**Background:**

Breast cancer (BC) remains a major global health concern, accounting for 11.7% of all cancer cases and ranking as the second leading cause of female cancer-related deaths worldwide. Increasing evidence highlights the interplay between gut microbiota (GM) dysbiosis and obesity-associated metabolic dysfunction in BC progression. This review aims to elucidate the role of GM in obese patients with BC.

**Methods:**

A systematic literature search was conducted in PubMed and Web of Science databases for publications from July 2015 to January 2025. Search terms combined BC, GM, obesity, dysbiosis, immunity, and microbiome. Article selection prioritized studies investigating microbial alterations in BC patients, mechanistic links between obesity and cancer progression, and GM-targeted interventions. Both original studies and authoritative reviews were included, supplemented by manual reference screening.

**Discussion:**

Obesity may trigger systemic inflammation, altered adipokine secretion, and disrupted steroid hormone metabolism *via* gut-derived β-glucuronidase activity, thereby exacerbating BC occurrence and recurrence. GM dysbiosis-driven metabolites such as branched-chain amino acids (BCAAs) and short-chain fatty acids (SCFAs) can activate oncogenic signaling pathways and immunosuppressive myeloid-derived suppressor cells (MDSCs), fostering tumor immune evasion. Conversely, dietary interventions, probiotics, and fecal microbiota transplantation (FMT) can alleviate dysbiosis, strengthen gut barriers, and restore anti-tumor immunity, improving chemotherapy response and reducing recurrence. However, challenges persist in deciphering BC subtype-related microbial signatures and optimizing microbiota-targeted therapies.

**Conclusion:**

Future longitudinal studies are needed to clarify causal relationships, validate microbial biomarkers, and translate preclinical findings into clinical applications. Addressing the gut-breast axis may offer transformative potential for precision oncology in obesity-driven BC.

## KEY MESSAGES

1. Systematic integration of gut microbiota, obesity, and breast cancer interactions.
2. Gut dysbiosis resulting from obesity exacerbates breast cancer via immune pathways.
3. Probiotics inhibit breast cancer, mitigate high-fat diet-induced obesity, and dietary modulation of gut microbiota metabolites improves breast cancer prognosis.

## 1. Introduction

Beginning in the mid-2000s, there has been a gradual rise in the incidence of BC among women, with an estimated annual increase at around 0.6% [1]. In the presence of the existing trends, the incidence of BC is projected to rise to over 3 million new diagnoses and 1 million fatalities annually by the year 2040, primarily attributable to demographic changes such as population expansion and an aging populace [2]. In particular, it occurs more frequently in women under 50 years of age (1.1% per year) than in those aged ≥50 years (0.5% per year), with the elevated incidence partially explained by declining fertility and increasing obesity [3].

Historically, cancer has been perceived as predominantly shaped by both host genetic factors and epigenetic modifications [4]. In the majority of cancers, the major contributing factors are spontaneous somatic mutations in critical oncogenes and tumor suppressor genes, which are modulated by an array of risk factors, including lifestyle choices, obesity, and exposure to carcinogenic substances. Nevertheless, a wealth of evidence in the last six decades has documented the pivotal roles of infections in cancer pathogenesis, with microorganisms being associated with roughly 13% of global cancer cases, translating to approximately 2.2 million new cases each year [5]. Critically, the GM constitutes a sophisticated ecosystem, made up of trillions of intestinal microorganisms, that maintain a symbiotic relationship with their host. Changes in the GM can lead to various health issues, as it can effect chemokine and cytokine synthesis, both of which are crucial for the regulation of the immune response. Moreover, microbiota is a major participator in the process of carcinogenesis (e.g. cancer initiation, progression, and metastasis) in the host, both positively and negatively [6]. Mechanistically, it can disrupt cell growth and apoptosis balance, engage genetic modifications, trigger inflammation, alter immune responses, and affect co-metabolism. Existing studies have documented established relationships of microbial dysbiosis with carcinoma involving the lung [7], breast [8], esophagus [9], stomach [10], colon and rectum [11,12], liver [13,14], biliary tract [15], pancreas [16–18], prostate [19] and cervix and uterus [20]. Changes in microbial species linked to cancers support the idea of a stable pathogenic microbiota with cancer-promoting traits.

In the past few decades, there has been a sharp increase in the proportion of people who are categorized as overweight (body mass index [BMI] 25-29.9 kg/m^2^) or obese (BMI ≥30 kg/m^2^) worldwide. A recent publication by the World Cancer Research Fund and the American Institute for Cancer Research highlights a strong link between adipose tissue buildup and various cancers, including esophageal adenocarcinoma, colorectal, pancreatic, postmenopausal breast, endometrial, and renal cancers [21]. It may be related to modifications in adipokine metabolism, localized inflammation, oxidative stress, and changes in immune response [22]. Moreover, patients suffering from obesity may exhibit an elevated overall mortality when diagnosed with breast, colorectal, or uterine cancers, as revealed by a comprehensive meta-analysis. Furthermore, individuals with breast, colorectal, prostate, and gastroesophageal cancers may also experience notable increase in the relapse [23]. Therefore, the present study was designed to characterize the GM in obese patients with BC and explore their relationship, as well as the impact on immunity, metabolism, and prognosis.

## 2. The role of GM in obese patients with BC

### 2.1. GM in BC patients

In general, BC patients may exhibit significant alterations in GM when compared to that of healthy individuals, suggesting a possible link of specific microbial populations with BC progression, and responses to treatment [8]. Microbial dysbiosis has been reported to be associated with numerous risk factors for BC, including genetic predisposition, psychological stress, dietary habits, levels of physical activity, lactation practices, mode of delivery, antibiotic usage, tobacco use, age, and alcohol intake [24]. For example, the α-diversity of the GM has been demonstrated to be significantly reduced in patients diagnosed with BC as opposed to healthy individuals, coupled with a notable increase in the relative abundance of *Firmicutes* compared to *Bacteroidetes* within this patient population [25]. Moreover, BC patients have remarkably lower levels of *Bifidobacterium*, *Shigella*, *Clostridium*, *Escherichia coli*, *Bacteroides uniformis*, *Faecalibacterium prausnitzii*, *Clostridium hathewayi*, *Akkermansia muciniphila*, and *Clostridium perfringens* compared to healthy females [26]. At the species level, cancer patients exhibit a reduced abundance of *Odoribacter* sp., *Butyricimonas* sp., and *Coprococcus* sp. in relative to healthy controls [27]. Notably, advanced-stage BC patients reveal higher detection rates of *Bacteroidetes*, *Clostridium coccoides*, *Clostridium leptum*, and *Blautia* species [28]. Furthermore, a study revealed that premenopausal BC patients had a significant increase in the abundance of *Bacteroides fragilis*, *Anaerostipes*, *Sutterella*, and *Haemophilus parainfluenzae* compared to healthy premenopausal individuals. In contrast, these patients showed a marked reduction in the abundance of *Bifidobacterium longum*, *Bifidobacterium bifidum*, *Bifidobacterium adolescentis*, *Faecalibacterium prausnitzii*, *Ruminococcus gnavus*, and *Rothia mucilaginosa* [29]. In addition, postmenopausal BC patients have been discovered with increased *Escherichia coli*, *Klebsiella*, *Prevotella amnii*, *Enterococcus gallinarum*, *Actinomyces spp. HPA0247*, *Shewanella putrefaciens*, and *Erwinia amylovora*, while reduced *Eubacterium eligens* and *Lactobacillus vaginalis* [8] compared to healthy postmenopausal individuals (Table 1).

**Table 1.** Alterations in GM associated with BC patients with different menstrual statuses.

BC patients with different menstrual statuses	Increased in GM	Decreased in GM	References
Postmenopausal BC Patients *vs.* Healthy Postmenopausal Individuals	- *s_Actinomyces spp. HPA0247 - s_Enterococcus gallinarum - s_Erwinia amylovora* *- s_Escherichia coli* *- s_Prevotella amnii* *- s_Shewanella putrefaciens* *- g_Klebsiella*	*- s_Eubacterium eligens - s_Lactobacillus vaginalis*	[8]
Premenopausal BC Patients *vs.* Healthy Premenopausal Individuals	*- s_Bacteroides fragilis* *- s_Haemophilus parainfluenzae* *- g_Anaerostipes* *- g_Sutterella*	*- s_Bifidobacterium adolescentis* *- s_Bifidobacterium bifidum* *- s_Bifidobacterium longum* *- s_Faecalibacterium prausnitzii* *- s_Rothia mucilaginosa* *- s_Ruminococcus gnavus*	[29]

Abbreviations: GM, gut microbiota; BC, breast cancer.

In recent years, some studies have revealed that the GM is specific in different subtypes of BC. In estrogen receptor-positive BC, the genera Adlercreutzia and Parabacteroides were identified as protective factors, whereas the genus Sellimonas was identified as a risk factor. Conversely, in estrogen receptor-negative subtypes, the genus Desulfovibrio demonstrated a protective effect, and Ruminococcaceae (UCG013) showed a suggestive protective trend. Mechanistically, high expression of CACNA1S, the functional gene associated with Adlercreutzia, correlated with a favorable prognosis in BC patients, whereas high expression of ERBB4, the functional gene associated with Sellimonas, predicted poorer outcomes [30]. In triple-negative breast cancer (TNBC), the GM primarily influences disease progression and responses to immunotherapy by modulating the immune microenvironment. Studies indicate that microbial species such as Akkermansia muciniphila, Bifidobacterium longum, Bacteroides fragilis, and the Ruminococcaceae family are associated with improved responses to immune checkpoint inhibitors (e.g. anti-PD-1/PD-L1 antibodies). Potential mechanisms involve the activation of dendritic cells, enhancement of CD8+ T cell anti-tumor activity through metabolites like short-chain fatty acids, and remodeling of the tumor immune microenvironment. In contrast, the relative abundance of Faecalibacterium and Clostridium has been correlated with poorer immunotherapy responses [31]. These findings suggest that interventions targeting the distinct gut microbial signatures of different BC subtypes, such as probiotic supplementation or dietary modulation, represent promising novel strategies for improving patient prognosis.

Besides, a recent research on human tumor microbiome revealed the presence of certain bacterial constituents across seven distinct types of solid tumors, namely breast, lung, ovarian, pancreatic, bone, skin, and brain cancers [32]. Every tumor displayed a distinct microbiome, and in particular, the microbiota associated with BC stood out in aspects of the abundance and diversity when contrasted with those found in other cancer types. The authors provided metabolomic insights. They noted that BC is marked by elevated oxidative stress. Furthermore, they found a significant proportion of bacteria that produce mycothiol, a compound which evidently detoxifies reactive oxygen species.

### 2.2. GM in obese people

The GM is recognized as a key factor in metabolic disorder onset, including obesity. It functions as an endocrine organ that significantly regulates host energy balance and immune responses. For instance, germ-free mice were observed without obesity signs, but obese mice developed such symptoms, highlighting the role of GM in obesity onset [33]. Furthermore, a population-based investigation revealed notable variations in the composition of GM from obese individuals when contrasted with the general populace [34]. Consumption of a high-fat diet (HFD) is confirmed to be linked to obesity-associated dysbiosis. This dysbiosis is characterized by a reduced total microbiota population, altered abundance of bacterial species, and increased gut permeability [35]. The genera *Staphylococcus* and *Clostridium*, belonging to the *Firmicutes* phylum, have been demonstrated to be positively correlated with obesity [36]. Critically, the *Firmicutes* phylum encompasses numerous species that are capable of producing butyrate, and elevated synthesis of butyrate and acetate may enhance energy extraction in individuals with obesity [36]. Moreover, acetate can be absorbed and utilized as a precursor for both lipogenesis and gluconeogenesis within the liver [37]. The *Bacteroides*, classified under the phylum *Bacteroidetes*, has been unveiled to have an inverse correlation with obesity in overweight and obese women suffering from metabolic disorders [38] following Roux-en-Y gastric bypass [39,40] and laparoscopic sleeve gastrectomy. *Bifidobacterium* under the *Actinobacteria* phylum, relates inversely with obesity across multiple populations, including pregnant women, children, and infants of normal weight mothers [36]. Species of *Bifidobacterium* possess the ability to deconjugate bile acids, a process that can potentially reduce the absorption of fats [41]. In addition to *Bacteroidetes* and *Firmicutes*, specific bacterial taxa, such as the *Christensenellaceae* family as well as the genera *Akkermansia*, *Bifidobacteria*, *Methanobacteriales*, and *Lactobacillus*, have also been discovered to be linked with obesity [42]. Recent studies link *Christensenellaceae* to weight reduction and gene expression in subcutaneous adipose tissues. Significantly, the relative prevalence of this family exhibited an inverse correlation with the BMI of the host [42,43]. Furthermore, while *Lactobacillus reuteri* and *Lactobacillus gasseri* exhibited positive associations with the prevalence of obesity, *Lactobacillus paracasei* demonstrated an inverse correlation. It supports a species-dependent association of bacteria with obesity, and different members within the same genus may influence obesity in distinct ways [44].

### 2.3. GM in obese patients with BC

**Figure 1.**
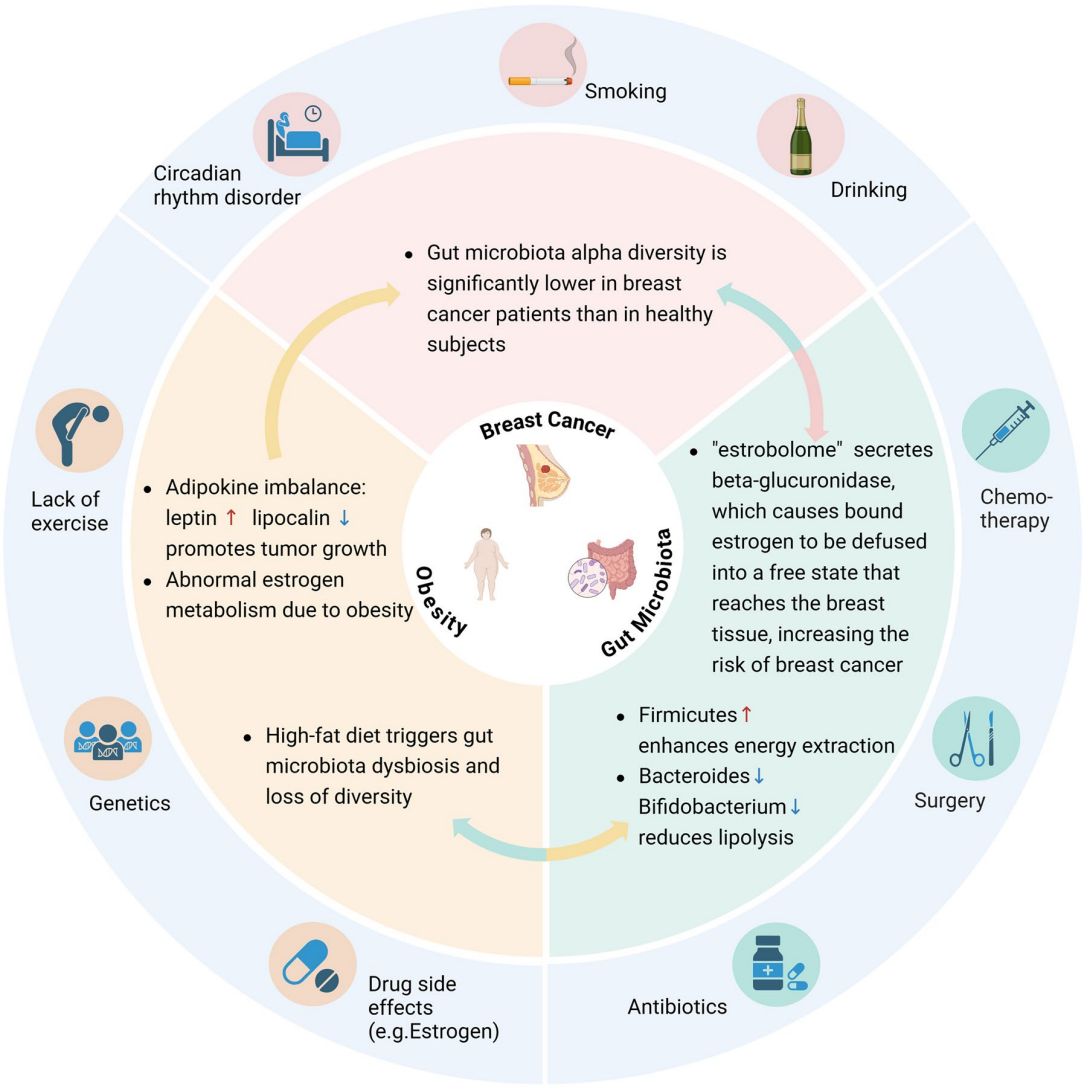
BC, GM, and obesity interact with each other. BC is affected by endogenous and exogenous factors, including smoking, drinking, stress, circadian rhythm disruption, and radiation therapy. Bad moods and unhealthy diets can also alter the GM. Breast tumors have a diverse microbiome within them. Stress, lack of exercise, genetics, drug side effects (e.g., estrogen), and high-fat diets can contribute to obesity. In obese populations, adipokine imbalance (e.g., increased leptin, decreased adiponectin) and disturbed energy metabolism—such as the regulation of GLP-1 and PYY secretion by SCFAs, which indirectly affect insulin sensitivity—are commonly observed. Both BC and obesity contribute to dysbiosis. In BC patients, the levels of bacteria such as *Clostridium coccoides* and *Escherichia coli* increase, while those of *Akkermansia muciniphila*, *Bifidobacterium*, and *Faecalibacterium* decrease. Obesity contributes to the development of chronic inflammation (e.g., elevated TNF-α and IL-6), an increased *Firmicutes*/*Bacteroidetes* (F/B) ratio, and a decrease in butyrate production. Abnormal estrogen metabolism in postmenopausal obese individuals exacerbates BC risk. Specific GM can also directly regulate estrogen metabolism, influencing the development of BC. BC, obesity, and dysbiosis all contribute to chronic low-grade inflammation and metabolic disorders, synergistically promoting disease progression. Created in https://BioRender.com.

Women who are overweight or obese have a higher risk of BC than those with a healthy weight [28]. Severe obesity may elevate the risk of BC-associated mortality by approximately 2.26 times [45]. BMI represents a major regulator of the composition of GM in BC patients. Specifically, individuals classified as overweight or obese patients exhibit a reduced total abundance of certain GM, including *Faecalibacterium prausnitzii*, *Firmicutes*, and *Blautia* spp., when compared to non-obese patients [46]. In another study on the alterations in increased adiposity-attributed GM, individuals diagnosed with either primary or metastatic BC have a higher abundance of *Akkermansia muciniphila* [47]. The GM can foster the synthesis of specific short-chain fatty acids (SCFAs), revealing established correlations with elevated levels of peptide YY [48], ghrelin, insulin, and glucagon-like peptide-1 (GLP-1) production [49]. GLP-1, modulated by the GM, can obviously regulate both food consumption and insulin release. Obese patient have been discovered to have reduced levels of this hormone when compared to individuals with non-obese individuals [50]. The levels of butyrate, which is generated by the GM, have been reported to be reduced in individuals with obesity [51]. This specific SCFA is essential for sustaining energy balance, as it can stimulate leptin production in adipocytes and increase the release of GLP-1 from L cells [52]. Leptin has been accepted to be an initial adipokine, a hormone that is specifically secreted by adipose tissues [53]. Leptin directly activates multiple key intracellular signaling pathways including JAK/STAT, MAPK, and PI3K/AKT by binding to its receptor leptin receptor, thereby driving proliferation, survival, migration, and invasive capacity in BC cells. Leptin synergistically activates epidermal growth factor receptor with insulinlike growth factor 1 or forms Notch, Interleukin-1, Leptin crosstalk outcome networks with inflammatory factors, collectively promoting malignant progression in triple-negative BC. Additionally, leptin interacts with the estrogen receptor alpha to enhance estrogen signaling pathways, thereby accelerating the development of hormone receptor-positive BC [54]. Additional adipokines identified subsequently include adiponectin, tumor necrosis factor-alpha (TNF-α), and interleukin-6 (IL-6), whose release have been observed to be linked to the proliferation of tumors [55] (Figure 1).

## 3. Mechanisms linking dysbiosis of GM to obesity, inflammation and BC development

Gut dysbiosis has demonstrated associations with both obesity and chronic low-grade inflammation. In other words, altered microbiota composition and reduced diversity can induce obesity by affecting energy balance and regulating fat storage. Simultaneously, dysregulated microbiota can also damage cell adhesion proteins, disrupt the epithelial barrier, increase intestinal permeability, and bring intestinal contents into contact with host peripheral tissues, leading to the secretion of pro-inflammatory cytokines by host cells. With the induction of chronic inflammatory response, it can eventually promote the progression of obesity-associated BC [56,57].

**Figure 2.**
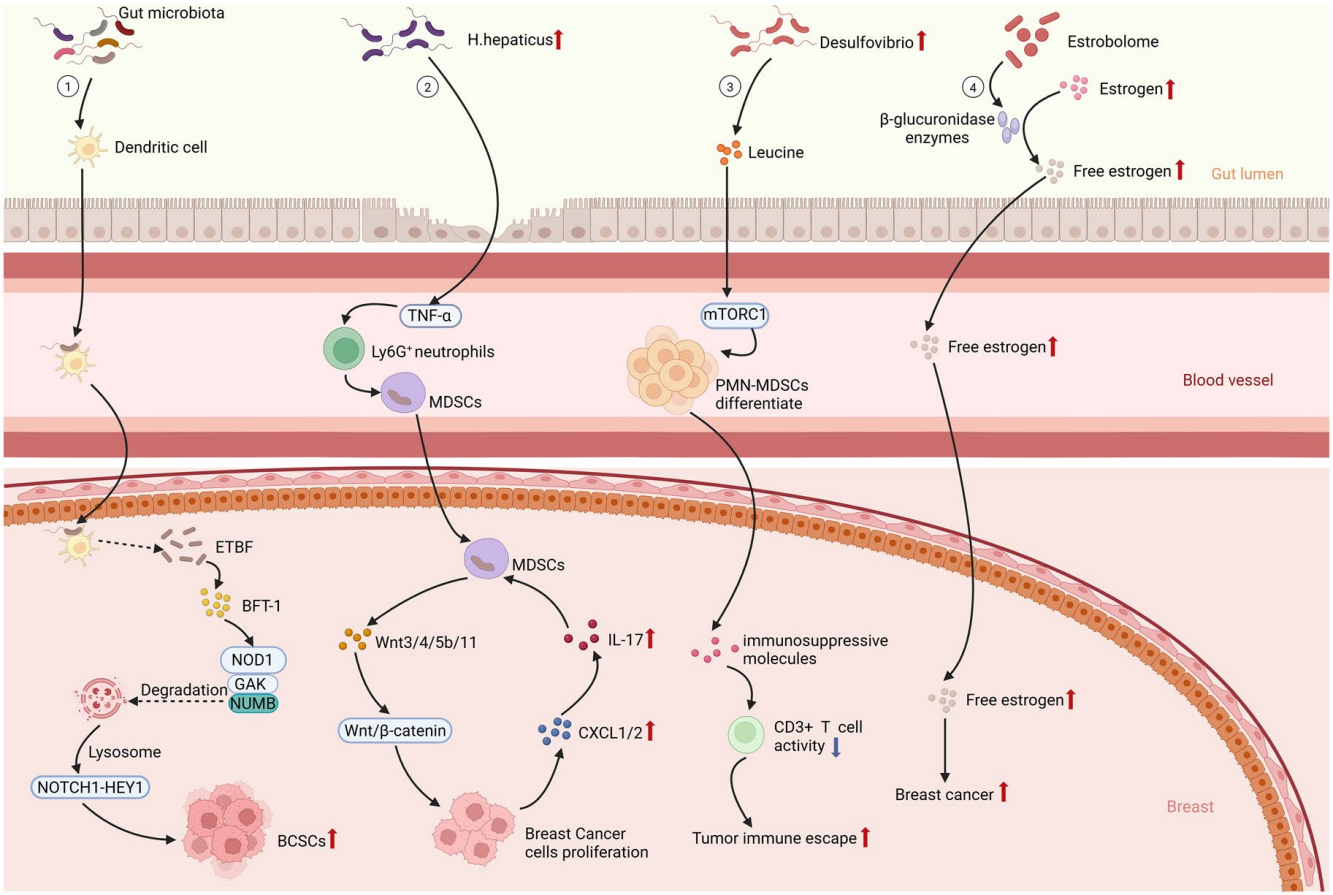
GM promotes BC through immune response. ① Bacteria in the gut are captured by dendritic cells. Dendritic cells can open the tight junctions of epithelial cells and subsequently transport bacteria via the vascular system to reach breast tissue. ETBF in breast tissue secretes the toxic protein BFT-1. BFT-1 directly binds to and stabilizes NOD1. NOD1 synergizes with GAK to phosphorylate NUMB, targeting NUMB for lysosomal degradation. This process consequently activates the NOTCH1-HEY1 signaling pathway, driving BCSCs propagation. ② *H. hepaticus* can activate TNF-α, thereby enhancing infiltration of Ly6G^+^ neutrophils and promoting the accumulation of MDSCs in breast tissue. These MDSCs enter the tissue and express Wnt3/4/5b/11 at high levels. They drive aberrant nuclear translocation of β-catenin in mammary epithelial cells, through activation of the Wnt/β-catenin signaling pathway, thereby inducing cellular proliferation and carcinogenesis. Meanwhile, CXCL1/2 chemokines released from breast tumor cell-associated stromal components bind to receptors induced by IL-17, mediating enhanced recruitment of MDSCs. ③ Desulfovibrio triggers the release of leucine through microbial metabolism, which activates the mTORC1 signaling pathway in bone marrow myeloid progenitors and drives the differentiation of PMN-MDSCs. PMN-MDSCs suppress CD3^+^ T cell activity, via immunosuppressive molecules, and promote tumor immune escape. ④The community of gut microbiota that influence estrogen metabolism and regulate the balance of circulating and excreted hormone levels is known as the estrogenome. They produce the enzyme β-glucuronidase, which hydrolyzes conjugated estrogen into free estrogen. The free estrogen then travels through the bloodstream and ultimately reaches breast tissue, increasing the risk of BC. Created in https://BioRender.com.

Recently, *Desulfovibrio*, a genus significantly enriched in the GM of obese BC patients (BMI > 24), has been noticed to be positively correlated with tumor size and the level of Ki67, a proliferation marker. Furthermore, in another study based on the construction of animal model, a HFD was shown to induce dysbiosis within the gut, which was marked by an increased prevalence of *Desulfovibrio*. This alteration in GM subsequently led to the liberation of branched-chain amino acids (BCAAs), leucine especially, as a result of microbial metabolism. Leucine, sourced from microbiota present in circulation, can engage the mTORC1 signaling pathway in myeloid progenitor cells located in the bone marrow, consequently facilitating the differentiation of polymorphonuclear myeloid-derived suppressor cells (PMN-MDSCs). Then, by suppressing CD3^+^ T cell activity *via* immunosuppressive molecules (e.g. S100A8/S100A9), PMN-MDSCs could boost tumor immune evasion to accelerate BC progression ultimately [58] (Figure 2).

The microbiome of the breast represents a specialized environment defined by specific microbial populations, their composition, and inherent properties [59]. Probiotics has been confirmed to be significantly effective in treating mastitis *via* oral administration, which is detectable in human milk [60], strongly implying a potential link between the GM and breast tissues. Its formation is hypothesized to result from bacterial movement from the areola and the entero-mammary route, where immune cells transport intestinal bacteria to lymph nodes and then to breast tissues [61,62]. Intestinal dendritic cells can internalize bacteria that disrupt epithelial tight junctions, illustrating an alternative evasion pathway [63]. Dendritic cells, based on their migratory nature, enable their movement toward remote locations, including mammary tissue, *via* the vascular network (Figure 2).

Meanwhile, the enterotoxigenic *Bacteroides fragilis* was examined to have higher levels in BC patients who were unresponsive to taxane chemotherapy. The finding particularly notable given that chemotherapy is known to disrupt the GM and alter its environment. Despite its minimal biomass, enterotoxigenic bacteroides fragilis (ETBF) released the toxic protein bacteroides fragilis toxin-1(BFT-1), facilitating the maintenance of the stemness in BC cells and contributing to their resistance to chemotherapy [64]. Mechanistically, BFT-1 could directly interact with nucleotide binding oligomerization domain containing 1 (NOD1) to stabilize the NOD1 protein. Notably, NOD1 exhibited elevated expression levels on aldehyde dehydrogenase breast cancer stem cells (BCSCs) and worked in conjunction with cyclin g-associated kinase (GAK) to phosphorylate NUMB, thereby promoting its lysosomal degradation. This process subsequently activated the NOTCH1-HEY1 signaling pathway, leading to an increase in the population of BCSCs [64] (Figure 2).

Additionally, obesity-associated systemic inflammation may exacerbate local immune responses induced by the involvement of *Helicobacter hepaticus*, a pathogenic organism residing in the liver and intestines of mice [31]. In a model of mice predisposed to mammary tumors, *H. hepaticus intestinalis* has been implicated in the enhancement of both mammary and intestinal tumorigenesis. Additionally, commensal bacteria within the gastrointestinal tract can disseminate to remote organs to advance cancer development. The dysbiosis from *H. hepaticus* can promote bacterial spread, allowing intestinal bacteria to migrate to the mammary gland, thus creating a pro-inflammatory environment [65]. In another animal study, *H. hepaticus* infection was detected to exert a carcinogenic effect, manifesting as increased tumor burden through a TNF-α-dependent pathway [66], which was closely linked to the activation and infiltration of Lymphocyte antigen 6 complex locus G6D^+^ (Ly6G^+^) neutrophils, as neutrophil depletion can markedly suppress tumorigenesis [66]. Furthermore, *H. hepaticus*-induced inflammation can promote the accumulation of MDSCs in mammary tissues. These MDSCs can highly express Wnt3/4/5b/11 and drive aberrant nuclear translocation of β-catenin in mammary epithelial cells by activating the Wnt/β-catenin signaling pathway, thereby inducing cellular proliferation and carcinogenesis. Concurrently, IL-17-expressing mast cells and the tumor microenvironment can secrete CXCL1/2 chemokines to recruit additional MDSCs, thereby forming a positive feedback loop that drives BC development [65] (Figure 2).

Indeed, the immunoregulatory mechanisms within the breast can be affected by the presence of a breast tumor, and this influence is further modulated by the consumption of fermented milk derived from *Lactobacillus helveticus* (*L. helveticus*). In a study involving mice, those injected with *L. helveticus* R389-fermented milk, in conjunction with breast tumor cell injections, exhibited highly reduced levels of IL-6 in serum and mammary gland, alongside a corresponding increase in IL10. This cytokine profile ultimately contributed to the inhibition of breast tumor cell proliferation [66]. The research used BALB/c (B Albino c) mice with induced BC to study the effects of *Lactobacillus acidophilus* (*L. acidophilus*) on immune responses. Their findings suggested that daily treatment with *L. acidophilus* led to an enhancement in the secretion of IL-12, a key immunomodulatory cytokine, within splenocyte cultures while concurrently attenuating tumor growth in the experimental mice [67]. Additionally, *L. helveticus* can ameliorate the effects of HFD-induced obesity in mice [68]. However, direct evidence of its carcinogenic effect on humans remains limited, and further exploration is needed in the future.

## 4. Association of GM and its metabolites with obesity combined with BC and intervention strategies

Probiotics are a group of active microorganisms that benefit the host, which can be categorized into three major groups, such as *Lactobacillus*, *Bifidobacterium*, and Gram-positive *cocci*. Probiotics, with extensive investigation regarding their capability, serve as a safe and effective option for supplying advantageous microbiota to a host organism [69]. The latest study defined probiotics as live microorganisms that, when administered in adequate amounts, confer a health benefit on the host [70]. Currently, widespread attention has been given to the potential of probiotics in combating obesity-associated BC.

As is known to all, the dysregulation of sex hormones, a hormonal imbalance, represents a significant risk factor for BC onset, which is evident in both clinical presentations and molecular characteristics across various subtypes of BC [71,72]. It has been established that estrogen metabolism and hormone balance can be affected by certain gastrointestinal microorganisms [73]. The estrobolome microbiota can synthesize beta-glucuronidase enzymes, modifying estrogens for better absorption into the bloodstream, after which the liver can process them before secretion into the gastrointestinal tract. Subsequently, bacterial β-glucuronidase can catalyze the de-conjugation of these compounds, resulting in their reabsorption as unbound estrogens *via* the enterohepatic circulation, which may facilitate the distribution of free estrogens to various remote organs, including the breast tissue [74] (Figure 2). β-glucuronidase levels are higher in nipple aspirate fluid from BC patients than those in healthy women [75]. Moreover, metabolites that exhibit estrogen-like properties may also be generated through oxidative and reductive processes within the gastrointestinal tract, as well as through the stimulated synthesis of growth factors that are responsive to estrogen, thus exhibiting carcinogenic properties. Notably, various β-glucuronidase-producing bacteria are found within the *Clostridia* class, including *Clostridium leptum* and *Clostridium coccoides*, as well as within the *Ruminococcaceae* family [28,76] and the *Escherichia/Shigella* bacterial group [77]. A clear positive association has been identified between the relative abundance of the *Clostridiales* order and the ratio of estrogen metabolites to their parent estrogens; and conversely, an inverse correlation was noted with the *Bacteroides* genus. Furthermore, the metabolism of estrogens in postmenopausal individuals has been verified to be linked to the diversity of fecal microbiota [78]. In fecal specimens from BC patients, *Streptococcus* levels were noticed to be significantly associated with the activity of β-glucuronidase/β-glucosidase, enzymes that enable the cleavage of estrogen glucuronide conjugates, thereby enhancing the recirculation of estrogen within the body [79]. Currently, a diet rich in fiber and polyphenols has been proposed to improve the survival of BC patients, especially in those with higher BMIs [66,80]. For example, dietary fiber has been documented to affect the diversity of GM and lower the activity of intestinal β-glucuronidase, thereby reducing the deconjugation and subsequent reabsorption of estrogens [81].

**Figure 3.**
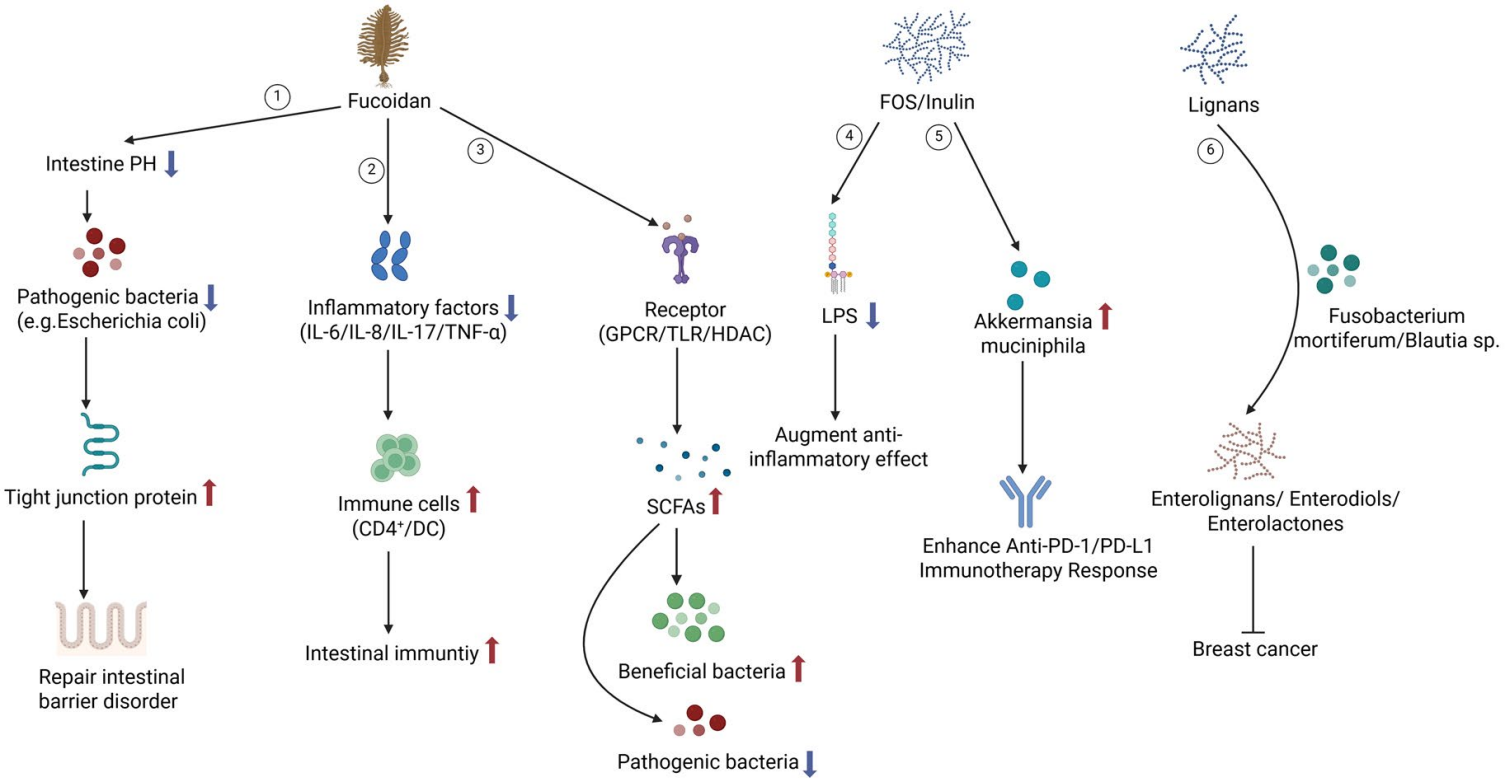
Probiotics and prebiotics inhibit BC through metabolism. ① Brown algae polysaccharide inhibits the growth of pathogenic bacteria (e.g. *Escherichia coli*) by lowering the pH of the intestinal microenvironment. It also prevents pathogens from adhering to intestinal epithelial cells and promotes the expression of tight junction proteins, thereby protecting the intestinal epithelial barrier. ②Brown algae polysaccharide directly induces the maturation of immune cells (e.g. CD4^+^ T cells, DC) and enhances intestinal immune function by decreasing the expression levels of inflammatory cytokines (e.g. IL-6, IL-17, TNF-α). ③Brown algae polysaccharide binds to receptors expressed in intestinal epithelial cells as ligands, promotes the production of SCFAs, stimulating the growth of beneficial bacteria, reducing the number of harmful microorganisms, and regulating the flora structure. ④ FOS and Inulin are able to reduce serum LPS levels, which enhance the anti-inflammatory effect. ⑤ FOS and Inulin promote the selective proliferation of beneficial bacteria such as *Akkermansia muciniphila*, which may enhance the response to Anti-PD-1/PD-L1 immunotherapy. ⑥ Plant lignans are converted into enterolignans, enterodiols, and enterolactoned by *Fusobacterium mortiferum* and *Blautia* sp., which in turn slow the growth of BC. Created in https://BioRender.com.

The intake of soy products, enabling the intake of isoflavones, may lower BC risk. A study in Japan showed an inverse relationship of miso soup consumption to BC incidence [82]. In another cohort study, soy and isoflavones would protect against BC in postmenopausal women [83]. A probiotic beverage that includes *Lactobacillus casei Shirota* and soy isoflavones have been identified to inversely affect the occurrence of BC [84]. The mechanism through which soy can prevent BC may be attributable to the dual estrogenic and antiestrogenic properties of soy isoflavones, notably genistein and daidzein. Additionally, *Lactobacillus*, a genus comprising Gram-positive bacteria, supported by their probiotic characteristics, can boost the anti-cancer activity of tamoxifen and other endocrine system-targeting drugs, potentially lowering the occurrence of estrogen receptor-positive BC. A study found that *Fucus vesiculosus* extract can inhibit estrogen receptor activation and estradiol synthesis in various female cancer cell lines [85]. Mice with BC administered fucoidan were detected with enhanced diversity and composition of the GM, contributing to a strengthened intestinal barrier, thereby aiding in the prevention of BC [86] (Figure 3). *Fusobacterium mortiferum* and *Blautia* sp. CAG-257 allow for the transformation of plant lignans into enterolignans, specifically enterodiols [87], and enterolactones. In addition, the administration of plant lignans, which is subsequently converted to enterolactone (ENL), or the direct use of ENL itself, has been demonstrated to impede or postpone the proliferation of BC [88] (Figure 3).

Fecal microbiota transplantation (FMT) with *Eubacterium rectale*, *Eubacterium eligens*, *Eubacterium ventriosum*, and *Collinsella aerofaciens* in humanized BC models can suppress tumor growth [89]. The genetic alteration of female mice through HFD-FMT, which included the microbes *Akkermansia muciniphila*, *Encephalitozoon intestinalis*, and *Muribaculum caecimuris*, was associated with an elevated risk of BC in this population. Administration of mucinophilic *Ackermannia* increased the count of tumor-infiltrating lymphocytes in post-FMT germ-free mice. The addition of these specific strains could potentially improve the efficacy of anti-tumor immune checkpoint blockade (ICB), suggesting a promising avenue for future clinical applications [90].

Prebiotics, typically classified as either fibers or polyphenols, are substances that cannot be digested by the host. These compounds exhibit various health advantages by providing nutrients that are preferentially utilized by the microorganisms residing within the host [70]. Frequently encountered prebiotics consist of fructooligosaccharides (FOS), inulin, and galactooligosaccharides (GOS) [70]. Prebiotics are found in foods such as asparagus and garlic, but FOS, GOS, inulin, and xylooligosaccharides at higher doses are often as supplements in research [91]. These substrates can enhance the abundance of *Lactobacillus* and *Bifidobacterium* [70,92]. In a mouse model of obesity, the administration of oligofructose resulted in a 40% reduction in circulating serum levels of lipopolysaccharides (LPS) over a 12-week period, highlighting its potential role in modulating systemic inflammation that may be triggered by circulating LPS [93,94] (Figure 3). The involvement of serum LPS has been detected in the metastasis of BC, underscoring the importance of reducing its concentrations in the bloodstream [95]. The supplementation of prebiotics have been studied in other cancers, despite no such report in BC treatment. In a murine model, inulin or mucin slowed melanoma growth by altering GM taxa and boosting anti-tumor immune responses [96]. Furthermore, inulin can inhibit colon cancer cell proliferationin a murine model [96]. *Akkermansia muciniphila* exhibited the most substantial enrichment in mice administered inulin, resulting in suppressed progression of colon cancer. Moreover, this bacterial species is correlated with the efficacy of therapeutic response to anti-PD-1/PD-L1 immunotherapy [96,97] (Figure 3). Altogether, prebiotics may boost beneficial microbes and reduce inflammation, aiding BC patients before, during, and after treatment, underscoring the necessity for further research on investigation its benefits more comprehensively.

## 5. The effect of GM intervention on the prognosis of BC patients undergoing immunotherapy

Individuals who have survived BC account for almost 50% of the 8 million women who are currently cancer survivors in the United States [98]. Approximately 30% of individuals diagnosed with BC may ultimately die, despite positive prognosis for long-term survival typically. Predominantly, these fatalities arise from disease recurrence after a variable duration of clinical remission that follows initial multimodal treatment. Consequently, ineffective prevention of tumor recurrence constitutes a major reason for a significant proportion of BC-related deaths [99]. In a manner akin to epidemiological findings that associate obesity with a heightened risk of developing primary BC, obesity may correlate with an elevated likelihood of BC recurrence [100–103], along with a 30-40% elevated likelihood of BC-related mortality [104,105]. Patients with obesity who are subjected to surgical procedures may experience an increased risk of developing anesthesia-associated complications, particularly significant when compared to non-obese BC individuals undergoing systemic interventions (e.g. chemotherapy, hormonal therapy, radiotherapy, etc.) [106].

In terms of the application of multiple anti-tumor treatment modalities, the primary purpose is to successfully eradicate malignant cells to achieve disease remission and minimize the likelihood of recurrences. Nearly all treatment options, despite significant advancement, exhibit toxicity towards healthy cells, resulting in a spectrum of adverse effects, some of which can jeopardize patient survival. Meanwhile, the GM also exhibit a profound interrelationship anti-cancer therapies [107]. Radiotherapy, chemotherapy, immunotherapy, and other therapeutic interventions can alter the microbiome of patients. Concurrently, the composition of this microbiome may affect the effectiveness of these treatments and the emergence of associated adverse effects [108]. A major challenge in BC management is addressing the adverse effects of and resistance to chemotherapy, with mechanisms poorly understood due to various clinical, biological, and psychosocial factors. Chemotherapy can disrupt microbial diversity, causing dysbiosis and gastrointestinal toxicity, with an established relationship of microbiome changes to long-term efICBfects in cancer survivors [109]. Nevertheless, there is a scarcity of research focusing on the relationship between chemotherapy for BC and its effects on the GM. In a prior research, women undergoing neoadjuvant chemotherapy experienced a notable rise in the detection rate of *Pseudomonas* spp., accompanied by reduced bacterial diversity within BC tissues, coupled with a diminished presence of *Prevotella* in tumor tissues of untreated patients [110].

Intracellular microbiota in tumors is a significant aspect of tumor biology, observed in various cancers, even with unclear roles. With the construction of an mammary specific polyomavirus middle T antigen overexpression mouse (MMTV-PyMT) model, it was found that the removal of intratumoral bacteria could decrease the possibility of lung metastasis, without any interference with the growth of the primary tumor. By enhancing resistance to fluid shear stress through actin cytoskeleton rearrangement, these bacteria would improve the survival of the host cells during metastasis. For instance, there existed difference in the main bacterial genera (*Staphylococcus*, *Enterococcus*, *Streptococcus*, and *Lactobacillus*) between bile tumors and normal tissues; and the delivery of specific bacterial strains from tumor microbiota could promote the metastasis in two different murine models [111].

A study indicated that the supplementation of *Lactobacillus johnsonii* and blueberry extracts (BB) led to considerable alterations in the diversity of GM and lipid metabolism [112]. *L. johnsonii* was found to increase serum levels of long-chain fatty acids (LCFAs) in all participants after consuming a HFD. Conversely, a reduction in the levels of LCFAs was recorded in the adipose tissue of animals subjected to the HFD with the administration of BB. Furthermore, all subjects with HFD exhibited diminished protein levels of sterol regulatory element-binding protein 1 and sterol regulatory element-binding protein cleavage-activating protein upon treatment with *L. johnsonii*. The presence of *L. johnsonii* in conjunction with BB significantly altered the GM diversity, particularly β-diversity. A notable decline in α-diversity was recorded in the ileum of animals on the HF diet supplemented with both *L. johnsonii* and BB, whereas an increase was evident in the ileum of subjects on a low-calorie diet supplemented with either *L. johnsonii* or BB. Therefore, the supplementation of *L. johnsonii* and BB resulted in significant alterations in GM diversity and lipid metabolism. Moreover, the response to ICB in subjects with malignancies has a significant association with the GM. A study indicated positive correlation of a more significant presence of the commensal bacterium *L. johnsonii* with the therapeutic efficacy of ICB [113]. The application of *L. johnsonii* or tryptophan-derived indole-3-propionic acid (IPA) can markedly enhance the efficacy of αPD-1 immunotherapy as facilitated by CD8 T cells. On a mechanistic level, *L. johnsonii* can work in conjunction with *Clostridium sporogenes* to synthesize IPA. This metabolite is essential in regulating the stem-like properties of CD8 T cells and facilitates the development of progenitor exhausted CD8 T cells by increasing H3K27 acetylation in the super-enhancer region of Tcf7 [113]. Additionally, IPA can enhance the responsiveness of ICB in BC.

## 6. Prospects and limitations

Recent studies reveal that vertical sleeve gastrectomy (VSG) potently enhances BC immunotherapy *via* GM remodeling. Specifically, VSG-enriched Clostridiales elevate circulating BCAAs, which activate invariant natural killer T cells. This mechanism synergizes with αPD-1 therapy, reducing tumor volume by 71% in mice. Importantly, oral BCAA supplementation mimicked this effect, reducing tumor burden by up to 74%, offering a non-invasive therapeutic strategy. These findings nominate microbial and metabolic biomarkers for TNBC patients resistant to current therapies [114]. Moreover, this research points to a sequential, microbiome-centric strategy for obese BC patients. Invasive approaches like FMT and bariatric surgery (e.g. VSG) first demonstrate that GM remodeling can potently enhance immunotherapy efficacy. The key translation lies in identifying the effector metabolites, such as BCAAs, which mediate these the anti-tumor effects of both FMT and VSG by activating anti-tumor immunity. Consequently, the clinical trajectory is advancing from invasive procedures toward non-invasive, precision supplementation with defined microbial metabolites. This evolution promises a safer and more scalable adjuvant therapy, potentially guided by microbial biomarkers for patient stratification. Future research can also focus on exploring more targeted regulatory measures. Among these, immune-modulators and phage therapy represent two promising avenues. Specific microbial metabolites, such as IPA, have been shown to enhance the efficacy of immune checkpoint blockade by epigenetically reinforcing stem-like properties of CD8+ T cells [113]. Furthermore, bacteriophages, as tools for precise editing of the gut microbiota, offer the potential to selectively deplete pro-carcinogenic bacteria (e.g. β-glucuronidase-enriched taxa) while preserving beneficial commensals [77,115]. Combining these novel strategies with existing microbiota-targeting approaches may open new avenues for the precision therapy of obesity-associated breast cancer.

Artificial intelligence (AI) and machine learning are transforming how we study the gut microbiome’s role in BC. First, algorithms like k-nearest neighbors and decision trees can classify samples using microbial and clinical data. This helps distinguish healthy individuals from patients effectively [116]. Beyond basic classification, algorithms like random forest can provide deeper insights. When combined with interpretability methods such as SHapley Additive exPlanations, they help uncover complex, non-linear relationships between specific bacteria, obesity, and cancer progression [117]. This approach reveals potential biological mechanisms. AI also supports clinical translation. It can stratify patient risk and guide personalized treatment strategies [116]. Additionally, integrating AI models from nutrition science and metabolomics could provide a more holistic view [118]. This may advance precision medicine in BC care. Most current microbiome studies rely on numerical data. However, the visual characteristics of fecal samples, such as color and consistency, are often overlooked. In the future, through standardized fecal image records and in combination with computer vision and deep learning, it may be possible to link these macroscopic features with the microbial composition. Such a multimodal approach could uncover new associations between stool appearance, key bacteria, and metabolic health. This could lead to non-invasive diagnostic tools for obesity-related BC and other conditions.

There are still some unresolved gaps in the clinical research of BC regarding the GM. A major challenge is the limited translatability of preclinical findings. Fundamental differences between murine and human gut physiology, immune systems, and microbiome composition complicate the extrapolation of results from animal models to human patients [119]. Furthermore, the current regulatory framework and evidence base for microbiota-based therapeutics are underdeveloped. Most commercially available probiotics are regulated as dietary supplements for gastrointestinal health, not as drugs for oncology applications. This creates a gap in robust clinical trial data supporting their safety and efficacy in modulating cancer therapy responses, such as to immunotherapy [120]. Finally, while innovative approaches using non-viable microbial derivatives—including bacterial metabolites and extracellular vesicles—represent a promising therapeutic avenue, they remain largely in the experimental stage. Their potential to overcome the safety and scalability limitations of live biotherapeutics requires further validation [115].

The regulation of BC by GM still has some limitations. Clinical studies exploring the relationship between the gut, GM, and BC are limited by several methodological constraints. A primary issue is the considerable inter-individual variability in GM composition, driven by genetics, diet, and environment. This variability challenges the development of one-size-fits-all interventions, as patient responses are likely heterogeneous [121]. Compounding this problem is the lack of standardization in microbiome science. Variations in sample collection, DNA extraction, sequencing protocols, and bioinformatic analyses across studies hamper reproducibility and direct comparison of findings. Many clinical investigations also suffer from inadequate sample sizes and insufficient control for key confounders. Factors such as dietary patterns, precise medication history, and environmental exposures are not always rigorously accounted for, potentially biasing the results. Underpinning these issues is an incomplete understanding of the mechanistic crosstalk between gut microbes, their metabolic outputs, and host tumor biology within the context of specific BC subtypes. This knowledge gap impedes the rational design of targeted interventions. Collectively, these limitations highlight the need for larger, well-controlled trials with standardized methodologies to establish robust evidence for microbiota-targeting interventions in BC care.

Due to the nature of a narrative review, the methodology of this study does not fully comply with the standards of systematic reviews, and there may be a certain degree of subjectivity in the article selection process, resulting in slightly lower reproducibility compared to systematic reviews; this limitation also provides a research direction for future relevant systematic reviews or meta-analyses.

## 7. Conclusions

BC remains a major global health challenge with rising incidence. The GM, obesity, and inflammation interact intricately in BC pathogenesis. There is increasing evidence that obesity-related microbiota changes play a critical role in BC prognosis, and modulation of this axis holds promise for future personalized treatments. This review summarizes GM alterations in patients, its role in obesity-related metabolic dysfunction, and mechanisms by which dysbiosis promotes tumor progression and immune evasion. Modulating GM through probiotics, prebiotics or fecal microbiota transplantation shows promise for improving outcomes, especially in obese patients. Future studies should define subtype-specific microbial signatures, validate microbiota-targeted therapies in rigorous trials, and explore dietary interventions targeting microbial metabolites. Increasing awareness of the interaction between the GM and BC will contribute to the development of more effective prevention and treatment strategies.

## Data Availability

Data sharing is not applicable to this article as no data were created or analyzed in this study.

## Glossary

AI: artificial intelligence
BFT-1: Bacteroides fragilis toxin-1
BB: blueberry extracts
BMI: body mass index
BCAAs: branched-chain amino acids
BC: breast cancer
BCSCs: breast cancer stem cells
GAK: cyclin g-associated kinase
ENL: enterolactone
ETBF: *Enterotoxigenic bacteroides* fragilis
FMT: fecal microbiota transplantation
FOS: fructooligosaccharides
GOS: galactooligosaccharides
GLP-1: glucagon-like peptide-1
GM: gut microbiota
HFD: high-fat diet
ICB: immune checkpoint blockade
IPA: indole-3-propionic acid
IL-6: interleukin-6
LPS: lipopolysaccharides
LCFAs: long-chain fatty acids
Ly6G^+^: lymphocyte antigen 6 complex locus G6D^+^
MDSCs: myeloid-derived suppressor cells
NOD1: nucleotide binding oligomerization domain containing 1
PMN-MDSCs: polymorphonuclear myeloid-derived suppressor cells
SCFAs: short-chain fatty acids
TNBC: triple-negative breast cancer
TNF-α: tumor necrosis factor-alpha
VSG: vertical sleeve gastrectomy.
